# MicroRNA-independent roles of the RNase III enzymes Drosha and Dicer

**DOI:** 10.1098/rsob.130144

**Published:** 2013-10

**Authors:** Timothy M. Johanson, Andrew M. Lew, Mark M. W. Chong

**Affiliations:** 1 The Walter and Eliza Hall Institute of Medical Research, Parkville, Victoria, Australia; 2 Department of Medical Biology, University of Melbourne, Parkville, Victoria, Australia; 3 St Vincent's Institute of Medical Research, Fitzroy, Victoria, Australia; 4 Department of Medicine (St Vincent's), University of Melbourne, Fitzroy, Victoria, Australia

**Keywords:** RNase III enzymes, Drosha, Dicer, microRNA, RNA processing

## Abstract

The ribonuclease III enzymes Drosha and Dicer are renowned for their central roles in the biogenesis of microRNAs (miRNAs). For many years, this has overshadowed the true versatility and importance of these enzymes in the processing of other RNA substrates. For example, Drosha also recognizes and cleaves messenger RNAs (mRNAs), and potentially ribosomal RNA. The cleavage of mRNAs occurs via recognition of secondary stem-loop structures similar to miRNA precursors, and is an important mechanism of repressing gene expression, particularly in progenitor/stem cell populations. On the other hand, Dicer also has critical roles in genome regulation and surveillance. These include the production of endogenous small interfering RNAs from many sources, and the degradation of potentially harmful short interspersed element and viral RNAs. These findings have sparked a renewed interest in these enzymes, and their diverse functions in biology.

## 2. The ribonuclease III family of proteins

**Figure 1.**
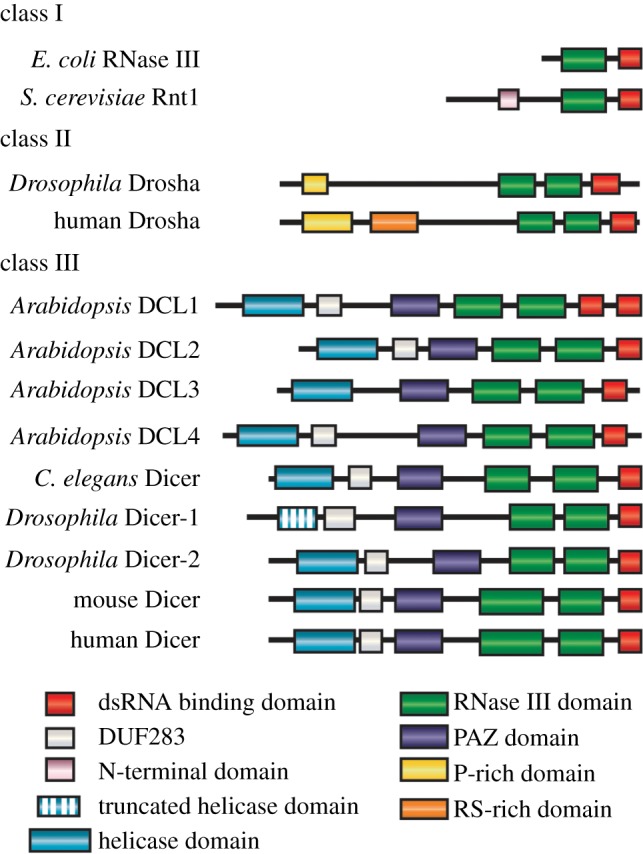
Structural characteristics of RNase III family members. The family is subdivided into three classes based on domain organization. Class I enzymes are found only in bacteria and simple eukaryotes, and are believed to be the antecedents of the more complex class II and III enzymes. All RNase III family members contain a dsRNA binding domain and RNase III domain, responsible for cleaving dsRNA. Evolution of these enzymes in higher eukaryotes led to the accumulation of additional domains. Of note is the acquisition of a helicase domain in many Dicer enzymes, which is likely to be important for unwinding dsRNA duplexes, and the PAZ domain, which binds to the 3′ end of target dsRNA. While in Drosha, proline-rich (P-rich) and/or arginine serine rich (RS-rich) domains are present in most species. The function(s) of these two domains is unclear, but they may function in protein–protein interactions.

Ribonuclease III (RNase III) family enzymes are found in virtually all eubacteria and eukaryotes, but not archaebacteria [1]. They are defined by characteristic RNase III domains, which, as dimeric modules, confer the unique ability to cleave double-stranded RNA (dsRNA). The family is divided into three classes based upon complexity (figure 1). Class I enzymes are the simplest, consisting of those found in bacteria and simple eukaryotes, such as RNase III in *Escherichia coli* and Rnt1 in *Saccharomyces cerevisiae*. These are thought to be the antecedents of the more complex class II Drosha and class III Dicer proteins. Class I enzymes achieve the dimeric catalytic RNase III module by forming dimers, whereas the more complex class II and III members use intramolecular dimerization of their two RNase III domains.

The ancestral RNase III members, such as bacterial RNase III, process ribosomal RNA (rRNA), whereas yeast RNase III is additionally able to process small nucleolar RNA [2,3], small nuclear RNA [4,5] and messenger RNA (mRNA) [6,7]. Until recently, it was thought that these abilities were not conserved in the more complex class II and III enzymes.

The class II Drosha and class III Dicer proteins are the best-known members of the RNase III family. This is due to their central roles in the biogenesis of microRNAs (miRNAs) and small interfering RNAs (siRNAs) in metazoans. miRNAs and siRNAs are families of small RNAs (19–24 nt) that regulate protein levels by targeting mRNAs for translational repression and degradation. However, it now appears that the functions of Drosha and Dicer go well beyond miRNA/siRNA biogenesis. This review will focus on the newly identified miRNA-independent RNA processing functions of these two enzymes, particularly in animals, and their importance in biology.

## 3. miRNA biogenesis

Drosha and Dicer are necessary for the biogenesis of most miRNAs. Canonical miRNA biogenesis begins with the transcription of a miRNA gene, typically by RNA polymerase II, which generates a long primary (pri)-miRNA transcript containing a short internal stem-loop structure [8,9]. This stem-loop structure is recognized by Drosha as part of a ‘microprocessor’ complex [10,11]. Guided by the dsRNA-binding protein Dgcr8 (known as Pasha in flies and nematodes) [12], Drosha cleaves the pri-miRNA at the base of the stem-loop, releasing it from the flanking single-stranded regions [13,14]. Cleavage of both arms of the stem-loop is dependent on the tandem RNase III domains of Drosha binding and cleaving the dsRNA stem. The released stem-loop structure is exported from the nucleus by exportin 5 [15,16–18] and is known as a pre-miRNA.

Once in the cytoplasm the pre-miRNA is cleaved by Dicer, in complex with another dsRNA-binding protein, Trbp [19,20]. The PAZ domain of Dicer binds the basal end of the double-stranded pre-miRNA, and guides the stem into a cleft formed by the intramolecular dimerization of two RNase III domains [21]. Scission of the RNA removes the loop structure, leaving a miRNA duplex. The distance from the PAZ domain to the RNase III domain dimer is thought to define the length of the RNA product, typically approximately 22 nt for miRNAs [22].

One strand of the miRNA duplex goes on to associate with the RNA-induced silencing complex (RISC), which it guides to target mRNAs. Facilitated by Argonaute proteins within the RISC, this targeting leads to translational repression, and ultimately degradation, of target mRNAs. The mechanisms of miRNA-mediated gene silencing have been reviewed extensively by others [23].

While the vast majority of miRNAs in metazoans are produced via this canonical pathway, a number of Drosha- or Dicer-independent miRNAs have been identified. The best-characterized are the mirtrons, which are dependent on splicing, rather than Drosha, for the pri- to pre-miRNA maturation step [24–26]. Numerous other Drosha-independent miRNAs have also been identified that are also independent of splicing [27–31]. However, their mode(s) of maturation have not been determined. Finally, the biogenesis of one miRNA, miR-451, has been shown to bypass Dicer, and is instead dependent on the catalytic activity of Ago2 [32–34]. Alternative miRNA biogenesis pathways have been reviewed elsewhere [35,36] and will not be discussed further.

## 4. Genetic evidence for miRNA-independent roles of Drosha and Dicer

There is a growing body of work demonstrating the importance of Drosha and Dicer in the processing of many classes of RNAs, in addition to miRNAs [37]. The recent development of mutant mouse models for Drosha or Dicer has accelerated the discovery of these miRNA-independent functions.

One of the early indications of miRNA-independent functions came from observations of differing phenotypes between Drosha- or Dicer-deficient cells. If both Drosha and Dicer are necessary only for miRNA maturation, then the assumption would be that deficiency in either enzyme would cause the same miRNA-dependent phenotype. While this has held true in most settings, there have been several prominent examples where Drosha or Dicer deficiency did not result in identical phenotypes. For example, Drosha deletion, but not Dicer deletion, in neuronal stem cells results in loss of stem cell fidelity and precocious differentiation [38]. By contrast, Dicer but not Drosha deficiency in the eye leads to macular degeneration [39,40]. These inconsistencies between the phenotypes caused by Drosha or Dicer deficiency indicated that the underlying mechanisms were independent of canonical miRNAs.

Transcriptional and proteomic analyses have reinforced the miRNA-independent activities of Drosha and Dicer. The transcriptional and proteomic changes caused by Drosha or Dicer deficiency have been shown to overlap only partially in a number of cell types, including T lymphocyte progenitors, neuronal progenitors and fibroblasts [29,38]. The differences between Drosha- or Dicer-deficient cells could be due to non-canonical miRNAs being particularly important in these cells, as is the case for the Dicer-independent biogenesis of miR-451 in red blood cell progenitors [32]. However, these differences could also be due to miRNA-independent functions of the two enzymes. These are discussed below.

## 5. miRNA-independent roles of Drosha

### 5.1. Cleavage of messenger RNAs

The best-characterized miRNA-independent role of Drosha is the recognition and cleavage of stem-loop structures in mRNA. The first mRNA identified as a direct cleavage target of Drosha was Dgcr8, another component of the microprocessor complex [41]. Drosha recognizes and cleaves stem-loop structures within the 5′ end of the Dgcr8 mRNA in mammalian cells, leading to destabilization of the mRNA. This cleavage therefore serves as a mechanism of gene repression, and is proposed to autoregulate the microprocessor complex [41]. The physiological relevance of this fine-tuning of Dgcr8 mRNA levels remains unclear. The cleavage of the Dgcr8 mRNA has since been reported to occur in many different cell types and species [29,42–47].

Although it was proposed that only the Dgcr8 mRNA is subject to direct cleavage by Drosha [46], it is now clear that this is not the case. The precocious differentiation of neural stem cells caused by Drosha deficiency is dependent, at least in part, on the derepression of the proneuronal transcription factor Neurogenin2 (Ngn2) [38]. In neuronal progenitors, Drosha normally binds and cleaves stem-loop structures within the 3′ UTR of Ngn2. However, in the absence of this regulation by Drosha, Ngn2 accumulates, resulting in a loss of stem cell fidelity and ultimately neuronal degeneration. This phenotype is independent of miRNAs, as miRNA levels were unaffected by Drosha deficiency within the time frame of the experiments. Furthermore, the phenotype was not reproduced with Dicer deficiency. Thus, this mRNA cleavage by Drosha, independent of miRNAs, has critical functions in biology.

The analysis of various Drosha-deficient cell types indicates that many other mRNAs are also subject to Drosha-mediated cleavage [29,43]. However, with the exception of Dgcr8 mRNA cleavage, this activity of Drosha appears to occur predominantly in stem/progenitor cell populations [29,38,43,46]. As such, non-redundant phenotypes caused by Drosha or Dicer deficiency occur most commonly in progenitor populations as opposed to mature, differentiated cell types [48–51].

The mRNA stem-loops that are recognized by Drosha appear to vary between cell types. For example, there are two stem-loop structures in the Dgcr8 mRNA, one of which is predominantly cleaved in some cell types, whereas the other is predominantly cleaved in other cell types [29,41,43]. This suggests that the activity of Drosha is modulated between different cell types or differentiation stages. This could be a result of different levels of Drosha being expressed, modification of mRNA secondary structure, or the presence or absence of cofactors required for the activity or affinity of Drosha. A number of factors have been shown to influence Drosha-dependent pri-miRNA cleavage, including p53, Lin28, DEAD-box RNA helicases and Smads [52,53]. This could also occur for mRNA substrates.

Similarly, cofactors are likely to be responsible for the considerable variation observed in the cleavage efficiency of different mRNAs. For example, Ngn2 mRNA is readily cleaved with at least 90% degradation by Drosha in neuronal progenitors, whereas there is only a twofold effect on Dgcr8 mRNA. In this context, the influence of cofactors is a particularly attractive notion because while Drosha is expressed in all cells, Ngn2 is not cleaved in all cells.

Differences in the processing efficiency of mRNAs versus pri-miRNAs by Drosha are also clear. Drosha appears far more efficient at processing pri-miRNA stem-loops than mRNA stem-loops [29]. Again, structure and/or sequence differences may dictate the efficiency of cleavage. Consistent with this, recent evidence indicates that there are species-specific sequence determinants within pri-miRNAs that regulate processing [54].

The stem-loop structures released following Drosha-mediated cleavage of mRNAs are occasionally processed further by Dicer to produce miRNAs [29,41]. However, because only vanishingly small quantities of these mRNA-derived miRNAs are ever produced (they can be detected only by high-throughput sequencing technologies), it is unlikely they are biologically functional. In recent years, high-throughput sequencing has identified that some 10% of human miRNA species appear to be derived from the exons of protein-coding and non-coding genes [55–57]. Given the low abundance of most of these, they may not be functional. However, these findings are consistent with the notion that many different mRNAs may be subject to cleavage by Drosha. Moreover, some 2000 protein-coding mRNAs in humans are predicted to contain some form of secondary stem-loop structure [58]. Thus, the potential physiological implications of Drosha-mediated cleavage are immense.

It also appears that direct mRNA cleavage by Drosha has been hijacked by at least one virus as a means of regulating viral gene expression. Karposi's sarcoma associated herpesvirus, like all herpesviruses, can shift between lytic and latent infection phases. Whether the infection is active or latent is associated with expression of viral Kaposin B [59]. Low-level expression is associated with latent virus infection, whereas the protein is highly expressed during viral replication and cell lysis. Mechanisms that regulate transcription of the Kaposin gene are important, but it now appears that cleavage of the mRNA by Drosha is also an important mechanism of repressing Kaposin levels during latency [60]. The cleavage of the Kaposin mRNA also results in the production of viral miRNAs that appear to be important in repressing other viral transcripts in order to reinforce latency [61]. Thus, at least one virus has commandeered Drosha's ability to cleave mRNA to its advantage.

### 5.2. Ribosomal RNA biogenesis

Even before the discovery of its requirement in miRNA biogenesis, it was suggested that Drosha is the metazoan orthologue of bacterial RNase III, an enzyme known to be involved in the maturation of bacterial rRNAs. Whether this ability is conserved in Drosha remains contentious. Wu *et al*. [62] showed that interference of Drosha by antisense RNAs affected rRNA processing in HeLa cells. More recently, this Drosha-mediated rRNA processing was implicated in regulating cell cycle progression in human multi-potent stromal cells [63]. Drosha knockdown, but not Dicer knockdown, in these cells led to a partial stall in the G1 phase of the cell cycle. While knockdown of Drosha or Dicer affected miRNA levels to a similar extent, only Drosha knockdown resulted in an accumulation of pre-rRNAs. Such a perturbation in rRNA biogenesis could potentially affect the G1/S phase transition [64,65]. Knockdown of other components of the Drosha microprocessor complexes, such as p68 and p72 DEAD-box helicases, also affects the levels of some rRNAs [66,67]. Cleavage of pre-rRNA by Drosha has been shown to occur, at least *in vitro*, and, similar to mRNA cleavage, the processing of these pre-rRNAs appears to be far less efficient than the processing of pri-miRNA [66].

Although the studies above reported various impacts on rRNA expression following Drosha knockdown [62,63,66], not all studies concur. For example, Lee *et al*. [9] observed only a block in miRNA maturation and not rRNA maturation following Drosha knockdown. More importantly, however, neither the targeted mutagenesis of *Drosha* [48] nor *Dgcr8* [68] in mice had any effect on rRNA expression. Why RNA interference (RNAi) of Drosha would have an effect on rRNAs, but a true knockout does not, is unclear. It is possible that the rRNA phenotypes may simply be artefacts of the RNAi. Thus, while ancestral RNase III members are capable of rRNA processing, it is unclear whether this ability has been preserved in Drosha [69,70]. If Drosha was necessary for the maturation of rRNA, one would imagine that loss of Drosha would have a catastrophic effect on cell survival, growth and proliferation. However, as discussed earlier, this is not the case, and Drosha deficiency results in the same phenotype as Dicer deficiency in the majority of cell types, indicating only miRNA-dependent impacts in these cells. In the few cell types in which Drosha deficiency causes a more severe phenotype, mRNA cleavage appears to be the more important lesion.

## 6. miRNA-independent roles of Dicer

### 6.1. Endogenous small interfering RNAs

Dicer is able to process highly diverse dsRNA structures in addition to pre-miRNAs [18]. The most widely studied is the biogenesis of siRNAs. First observed as unexpected gene repression in plants, fungi and worms following the introduction of transgenes [71–73], it was soon determined that siRNAs originating from dsRNAs (concatemers of transgenes are one source of these dsRNAs) were responsible for the phenomenon of RNAi [74]. It was subsequently determined that Dicer was responsible for the generation of these siRNAs from dsRNAs, and thus the overlap with miRNAs became apparent [19,75–77]. RNAi/siRNA is now of course widely used as an experimental tool for the knockdown of genes of interest.

However, siRNAs are not just experimental tools, but are also produced from endogenous sources. These endogenous siRNAs (endo-siRNAs) are derived from numerous sources of dsRNA, including small nuclear RNAs [78] and small nucleolar RNAs [30,31,79]. Dicer will also process viral sources of dsRNAs to produce viral siRNAs. These are involved in various anti-viral silencing responses [80]. Dicer processes these endogenous/viral dsRNAs in the same manner as pre-miRNAs, in that the PAZ domain binds the basal end of the structure and the tandem RNase III domains cleave both strands of the dsRNA [21]. Pairs or arrays of Dicer complexes dice the dsRNA at regular intervals, leaving siRNA duplexes of approximately 22 bp. Endo-siRNAs are important in the regulation of genome stability and gene expression. As outlined below, they achieve this either at a post-transcriptional level by targeting RNA for degradation, or at a transcriptional level by initiating and maintaining heterochromatin.

Mobile genetic elements litter the genomes of all animals. Movement of these elements can be genotoxic and cytotoxic. However, multiple mechanisms are in place to suppress their activity [81,82]. For example, post-transcriptional silencing of mobile genetic elements via RNAi has been demonstrated in both lower organisms and mammalian cells [83]. Analysis of small RNAs associated with *Drosophila* Ago2 determined that mobile genetic elements are a substantial source [84,85], whereas in human cell lines, it was shown that siRNAs derived from mobile genetic elements can return to target these same elements [86]. Mammalian oocytes also express endo-siRNAs from dsRNA derived from the concurrent transcription of gene–pseudogene pairs. Similar to mobile genetic element-derived endo-siRNAs, these appear to regulate the levels of the parental gene via RNAi [87–89].

The mechanisms of post-transcriptional silencing of mobile genetic elements in animals may have evolved from epigenetic mechanisms to silence transcription (as opposed to post-transcription) in single-cell eukaryotes. These are particularly well described in the fission yeast *Schizosaccharomyces pombe*, in which initiation and maintenance of heterochromatin is necessary for controlling the expression of certain loci. Although miRNAs are not found in simple eukaryotes, orthologues of some RNAi machinery components are present, including Dicer and Ago1. Nuclear siRNAs corresponding to the heterochromatic loci have been shown to correlate with silencing, whereas Dicer and Ago1 mutations disrupt the production of these endo-siRNAs and the assembly of heterochromatin [90,91]. By recruiting histone-modifying enzymes that initiate heterochromatin formation, these nuclear siRNAs induce sequence-dependent silencing via the RNA-induced initiation of transcriptional gene silencing (RITS) complex, consisting of Ago1, Chp1 and Tas3 [91,92]. Transcriptional silencing phenomena involving RNAi machinery components have been reported in *Arabidopsis* [93,94], *Drosophila* [95] and mammalian cells [96]. In the case of mammals, histone deacetylases and DNA methyltransferases have been shown to be important [96].

Ago and chromodomain proteins, such as Chp1, are present throughout eukaryotes. However, Tas3, a key component of the RITS, was identified as a protein restricted to fission yeast [97]. No orthologue has been identified in multicellular organisms. As such, it is still unclear whether this precise mechanism of endo-siRNA-dependent heterochromatization has been conserved through evolution, or whether additional transcriptional silencing mechanisms have evolved to use RNAi machinery components. Characterization of the RNA-dependent transcriptional silencing complex in higher organisms is key to deciphering this puzzle.

Dicer-dependent endo-siRNAs also have a role in triplet repeat expansion diseases. Genes mutated in these diseases, such as Huntington's disease and fragile X disorder, frequently encode RNAs with long internal triplet repeat structures. Dicer is able to recognize and process these structures into siRNAs [98]. This function staves off pathology triggered by the aberrant accumulation of the repeat structures, which cause protein disruption via toxic neuronal nuclear aggregates [99]. The siRNAs produced from the processing of these RNAs are thought to target further triplet repeat transcripts for degradation via the RISC. Thus, Dicer has direct and indirect roles in curtailing the amount of toxic triplet repeat RNAs.

### 6.2. Processing and detoxification of repeat-element-derived RNAs

Potentially harmful mobile genetic elements litter the genomes of higher eukaryotes, and multiple mechanisms have evolved to suppress their activity. Some elements are silenced post-transcriptionally by endo-siRNAs, as described earlier. Another example is the 3′ repair exonuclease Trex1, which binds and degrades ssDNA derived from a variety of mobile genetic elements [100]. Mutations in Trex1 in humans are responsible for the autoimmune diseases Aicardi–Goutières syndrome and chilblain lupus, owing to the accumulation of mobile genetic elements.

Cleavage by Dicer is a further mechanism for detoxifying repeat-element-derived transcripts, such as from short interspersed elements (SINEs). Geographic atrophy, a severe macular degenerative disease, was recently found to be associated with the downregulation of Dicer in humans [39]. Associated with this loss of Dicer is the aberrant accumulation of RNAs from *Alu* elements. A similar macular disease is recapitulated in mice by inactivation of the *Dicer1* gene specifically within the retinal pigment epithelium (RPE). This is accompanied by accumulation of RNAs from *Alu*-like B1 and B2 SINEs. Inactivation of other miRNA machinery components does not recapitulate the phenotype, indicating that the function of Dicer in the RPE is independent of miRNAs.

The accumulation of SINE RNAs was demonstrated to be the cause of the cytotoxicity in RPE cells as ectopic expression of *Alu*/B1/B2 RNAs is cytotoxic and recapitulates the damage caused by Dicer deficiency, whereas the Dicer phenotype is rescued by knockdown of these RNAs with antisense oligos. Dicer appears to detoxify SINE RNAs by degradation into small 25–50 nt RNAs. These are larger than the typical approximately 22 nt of miRNAs and siRNAs. Whether these 25–50 nt products are functional in the RPE is unclear. The fact that interference of B1/B2 RNAs is sufficient to rescue the Dicer phenotype, and ectopic expression recapitulates the phenotype, suggests that it is the degradation of the RNAs that is most important in this setting.

These longer siRNAs produced by Dicer processing of *Alu* RNAs, however, do appear to have functions in neuronal stem cells [101]. In these cells, retinoic acid-induced *Alu* RNAs are also processed into siRNAs of the longer variety. Knockdown of Dicer perturbs neuronal stem cell proliferation and is accompanied by the loss of these siRNA, whereas Drosha knockdown does not. Referred to as repeat-induced small RNAs, these longer siRNAs appear to interact with Ago3 and decapping complexes to target specific mRNAs for translational repression and degradation in an analogous manner to miRNAs. A similar role for Dicer-dependent SINE-derived siRNAs was suggested in mouse embryogenesis; however, no specific mRNA targets were identified [102].

### 6.3. A death-promoting DNase

A hallmark of apoptosis, or regulated cell death, is the initiation of DNA breaks. Multiple DNases have been implicated in this process, including DNase 40-kDa DNA fragmentation factor (DFF40) [103]. In *C. elegans*, it has been shown that Dicer also plays a role in the recognition and processing of DNA [104].

This unexpected DNase function of Dicer is the result of conversion following cleavage by the caspase CED-3. This cleavage occurs within the first of the two RNase III domains of Dicer, leaving a truncated though catalytically active protein consisting only of the C-terminal RNase III and dsRNA-binding domains. Curiously, this truncation removes the ability of Dicer to process dsRNA, but imparts a new and unexpected DNase capability. Mechanistically similar to the processing of dsRNA structures, the remaining RNase III domain of this truncated Dicer can bind to and nick one strand of dsDNA. This activates DNA degradation and subsequent apoptosis. Deletion of other proteins essential for processing of small RNAs by Dicer does not impact DNA fragmentation in apoptosis, consistent with the miRNA-independent nature of this activity.

It is not clear whether this role of Dicer occurs in other organisms or is a nematode-specific phenomenon. Annotation of the *C. elegans* genome has yet to identify a DFF40 analogue [105], and perhaps Dicer plays the role of initiator of DNA fragmentation in lieu of DFF40 or other DNases. Further studies are clearly warranted to determine whether this miRNA-independent function of Dicer is conserved in other species.

## 7. Drosha and Dicer in the biogenesis of DNA-damage-associated small RNAs

In addition to the regulated DNA fragmentation associated with apoptosis, a myriad of genotoxic insults continuously induce DNA double-stranded breaks in cells, and multiple mechanisms are in place to repair this damage. Homologous recombination and non-homologous end-joining are two distinct mechanisms that have evolved to repair DNA breaks. Components of both repair pathways appear to be regulated by a series of miRNAs [106]. These miRNAs are thought to regulate the choice of which repair pathway is engaged in response to different insults. However, another class of small RNA has also been implicated in the DNA-damage response. These too are dependent on Drosha and Dicer for their biogenesis.

DNA-damage-induced small RNAs were first observed in the fungus *Neurospora crassa*. DNA lesions in *Neurospora* induce the transcription of precursor aberrant RNA (aRNA) from the ribosomal DNA locus [107]. This transcription is dependent on the DNA/RNA-dependent RNA polymerase QDE1 and a helicase QDE3, but is independent of RNA polymerase I–III. These precursor aRNAs are processed by Dicer-like proteins to generate approximately 21 nt RNAs that then associate with QDE2. The function of QDE-associated small RNAs still remains to be determined, but it is proposed that they may inhibit rRNA biogenesis and protein synthesis after DNA damage.

DNA damage also induces the production of non-miRNA small RNAs in more complex organisms, including plants, flies and vertebrates. The production of these DNA-damage RNAs (DDRNAs) or double-stranded break-induced RNAs (diRNAs) appears to be evolutionarily conserved between plants and animals. Both Drosha and Dicer are required for the biogenesis of these DDRNAs in animals [108], whereas their homologues, DCL1–4, all appear to contribute to diRNA production in plants [109].

Knockdown of either Drosha or Dicer in human cells permits aberrant DNA replication and cell division in DNA-damage-induced senescent cells [108]. Proper formation of DNA damage foci and senescence appears to be dependent on these DDRNAs. Unlike in *Neurospora*, these small RNAs appear to be derived from sequences near the dsDNA break itself. The same origin of diRNAs also occurs in plants [109]. The miRNA-independent nature of the DNA damage response in human cells was demonstrated by blocking proteins essential for miRNA-mediated translational repression, which had no impact on the proper formation of DNA damage foci.

The precise mechanism by which these small RNAs contribute to the DNA damage response is still unclear. One possibility is that DDRNAs/diRNAs function in an analogous manner to nuclear siRNAs in fission yeast, guiding chromatin-modifying complexes to sites of DNA damage. The resulting transcriptional silencing could potentially minimize further damage that may be caused by RNA polymerases blocking the repair machinery [110]. Because of sequence homology, it is also possible that DDRNAs/diRNAs guide DNA repair machinery to the site of damage in a manner similar to miRNAs/siRNAs guiding the RISC to target mRNAs for silencing.

If these DDRNAs/diRNAs are to be generated by Dicer- and Drosha-mediated processing, their precursors must form dsRNA structures. In *Arabidopsis*, it has been proposed that a single-stranded transcript is generated by resection of the DNA damage site, which then acts as a template for RNA-dependent RNA polymerase (RDRP) to generate a dsRNA [109]. This dsRNA precursor would then be a substrate of one of the DCL proteins. However, the mechanism that generates the RNA precursor in animals is still unknown, given the absence of RDRPs. Sense and antisense transcription has been proposed as a mechanism of producing the dsRNA substrate [108,111], but evidence supporting this has yet to be found. The fact that Drosha is known to process stem-loop structures, rather than just any dsRNA, suggests a single-stranded nature to the putative DDRNA/diRNA precursor. Regardless of the mechanism, it is clear that RNase III enzymes, independent of miRNAs, play important protective functions in the response of cells to DNA damage.

## 8. Conclusion

**Figure 2.**
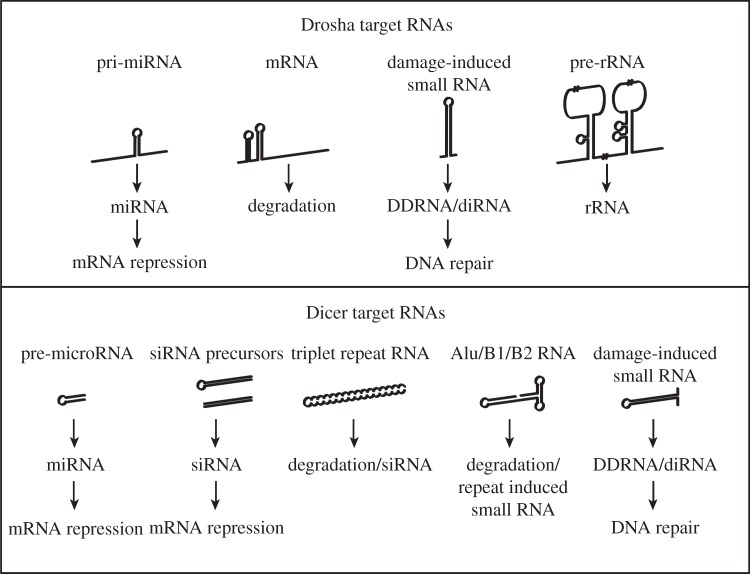
dsRNA substrates of Drosha and Dicer. dsRNA structures that can be recognized and processed by the RNase III enzymes Drosha and Dicer. Drosha cleaves various stem-loop structures similar to pri-miRNAs, such as within mRNAs, DNA-damage-induced RNAs and possibly pre-ribosomal RNA (pre-rRNA). Dicer can cleave a large number of different substrates with internal double-stranded structures, in addition to pre-miRNAs. These include endogenous dsRNAs to produce siRNAs, triplet repeat RNAs, SINE-derived RNAs and DNA-damage-induced RNAs.

From the earliest characterization of Drosha and Dicer proteins, it has been suggested that their structure would enable them to be involved in various RNA metabolic processes [19,62,75–77]. Since the discovery of their central roles in miRNA biogenesis, much focus has been placed on this function. However, a resurgent interest in the broader roles of these two RNase III enzymes has elucidated a plethora of functions in the processing of many biologically important non-coding and protein-coding RNAs (in the case of Drosha; figure 2). It is now clear that Drosha and Dicer are more aligned with their antecedent enzymes, and are far more versatile than was believed for many years. This versatility is proving to be important in a broad range of biological processes, and critical for maintaining cellular homeostasis and the prevention of pathology, such as those that occur in diseases from macular degeneration to triplet repeat diseases to cancer. Our understanding of these miRNA-independent functions of Drosha and Dicer is limited, and much work is still needed to elucidate the various mechanisms. Regardless, it is clear that RNase III enzymes play many important roles in biology, far more than simply in the biogenesis of miRNAs.
